# Intervalos de referencia de parámetros hematológicos en población chilena adulta y en la etnia mapuche

**DOI:** 10.1515/almed-2025-0014

**Published:** 2025-02-19

**Authors:** Pablo J. Letelier, Carolina A. Chicahual, Nicolás F. Arroyo, Daniel P. Monsalves, Rodrigo E. Boguen, Neftalí H. Guzmán

**Affiliations:** Departamento de Procesos Diagnósticos y Evaluación, Facultad de Ciencias de la Salud, Laboratorio de Investigación en Salud de Precisión, Universidad Católica de Temuco, Temuco, Chile

**Keywords:** etnicidad, hemograma, parámetros hematológicos, valores atípicos, intervalos de referencia

## Abstract

**Objetivos:**

Los intervalos de referencia (IR) son una herramienta esencial para apoyar la toma de decisiones clínicas. Estos pueden presentar variaciones intra e interindividuales asociadas a diferencias genéticas y factores medioambientales. Dado que la población de Chile está compuesta por múltiples grupos étnicos, estas variables adquieren aún mayor relevancia. El objetivo del presente estudio es establecer IR para diferentes parámetros hematológicos en la población chilena y la etnia mapuche.

**Métodos:**

Se seleccionó una muestra de 356 adultos (entre 18 y 65 años), de los cuales 146 pertenecían a la etnia mapuche, utilizando el método indirecto a posteriori a partir de la base de datos del laboratorio clínico UC Temuco. El análisis se realizó considerando el sexo y la etnia. Los valores atípicos se detectaron mediante la prueba de Tukey, mientras que los IR se establecieron aplicando el método no paramétrico recomendado por la IFCC.

**Resultados:**

La mediana de edad de la muestra global de la población general fue de 35 años en mujeres y 36 años en hombres. Se observaron diferencias estadísticamente significativas (p<0,05) por sexo en los parámetros dependientes de la hemoglobina y del recuento de plaquetas. En el análisis por etnicidad, se observaron diferencias significativas en el recuento de glóbulos rojos, hemoglobina y hematocrito (p<0,0001).

**Conclusiones:**

Este estudio demuestra que los intervalos de referencia hematológicos varían según el sexo y la etnicidad, lo cual debería ser considerado en una población multiétnica. Este hallazgo contribuye a un mejor conocimiento de las características individuales de cada persona, facilitando una interpretación clínica más precisa.

## Introducción

Los intervalos de referencia (IR) son los valores comprendidos entre los límites de referencia superior e inferior [1], y describen la variación de parámetros biológicos en individuos con características específicas (individuos de referencia) [2]. De este modo, los IR son esenciales en la toma de decisiones médicas, facilitando el diagnóstico, el monitoreo y la vigilancia epidemiológica de diferentes patologías humanas. Por lo general, los IR de las pruebas de laboratorio clínico no se suelen analizar en diferentes subpoblaciones ni se suelen basar en perfiles epidemiológicos o clínicos específicos, lo cual supone una limitación a la hora de interpretar los resultados de las pruebas analíticas [2], 3]. El hemograma es una de las pruebas analíticas más demandadas, ya que forma parte de los estudios básicos requeridos para obtener una orientación diagnóstica y evaluar el estado de los pacientes. Su relevancia se ha mantenido en el tiempo, habiendo evolucionado con la automatización del recuento celular y la incorporación de nuevos parámetros, como la amplitud de la distribución eritrocitaria (*red cell distribution width*, RDW) y la amplitud de la distribución plaquetaria (*platelet distribution width*, PDW) [4]. Los parámetros cuantitativos del hemograma, junto con el estudio de los índices eritrocitarios – como el volumen corpuscular medio (VCM), la hemoglobina corpuscular media (MCH) y la concentración de hemoglobina corpuscular media (MCHC), además del RDW, apoyan las interpretaciones clínicas en diversas patologías (poliglobulia, policitemia, anemia, etc.). Es frecuente hallar alteraciones en variables específicas, como neutropenia en pacientes que están recibiendo quimioterapia o que presentan infecciones o enfermedades inmunológicas, y neutrofilia como respuesta a enfermedades infecciosas, trastornos inflamatorios y neoplasias hematológicas [4], 5]. Además, el uso de cocientes como el índice neutrófilo-linfocito (*neutrophil-to-lymphocyte ratio*, NLR) y el índice plaqueta-linfocito (*platelet-to-lymphocyte ratio*, PLR) han permitido obtener más información clínica, ya que ambos están asociadas con el estado inflamatorio del organismo [6].

Debido a la dificultad de medir los IR, los laboratorios suelen emplear los valores proporcionados en las fichas técnicas de los fabricantes de reactivos o los establecidos en la literatura científica. El empleo de IR externos puede dificultar la correcta interpretación y manejo de los pacientes. La evidencia demuestra diferencias significativas en los IR entre diferentes grupos étnicos [7], con variaciones según el sexo, el área geográfica [8] y la edad [9]. La Ley Chilena 19,253 reconoce nueve pueblos indígenas [10], que representan el 12 % de la población nacional (2.185.732 habitantes), siendo el pueblo mapuche el más numeroso, ya que representa el 79,18 % de toda la población indígena del país (aproximadamente 1.745.147 personas). La región de La Araucanía, ubicada en el sur de Chile, es una de las siete regiones con mayor población indígena, albergando el 34,35 % de la población indígena, lo que equivale a unas 314.174 personas [10].

A pesar de la elevada heterogeneidad social, existen pocos estudios en Latinoamérica sobre los IR en las poblaciones locales [11]. Los estudios disponibles suelen estar limitados a características específicas, como la altura [12]. En Chile, únicamente se han publicado dos artículos para establecer IR del hemograma, uno sobre reticulocitos en una población pediátrica sana [13], y otro en el que se analizó una muestra de personal militar expuesto a una altitud elevada [14]. Dada la escasez de datos, es importante determinar los IR para la población de la región de La Araucanía, especialmente teniendo en cuenta que la población mapuche presenta patrones epidemiológicos de transición prolongada, caracterizados por una incidencia elevada de infecciones y enfermedades degenerativas crónicas [15]. Por tanto, el objetivo del presente estudio fue establecer IR para diferentes parámetros hematológicos en la población adulta chilena y en la etnia mapuche.

## Materiales y métodos

### Diseño del estudio

Se trata de un estudio retrospectivo, no experimental, con análisis univariado y multivariado. Los datos se obtuvieron del Laboratorio Clínico UC Temuco, ubicado en la ciudad de Temuco (Región de La Araucanía, Chile). Se seleccionó una muestra de 356 adultos (50 % hombres y 50 % mujeres) aplicando el método indirecto *a posteriori*, siguiendo las recomendaciones de la Federación Internacional de Química Clínica y Medicina de Laboratorio (*International Federation of Clinical Chemistry and Laboratory Medicine*, IFCC), que establece un número suficiente de individuos (≥120) para aplicar el método no paramétrico. De esta muestra, 146 participantes pertenecerían a la etnia mapuche, y se realizó un estudio comparativo con los participantes que no pertenecían a dicho grupo. Se analizaron los siguientes parámetros hematológicos en un analizador Sysmex Xs1000i (Sysmex Corporation, Japón): recuento de leucocitos (WBC), recuento eritrocitario (RBC), concentración de hemoglobina (HGB), hematocrito (HCT), volumen corpuscular medio (VCM), hemoglobina corpuscular media (MCH), concentración de hemoglobina corpuscular media (MCHC), RDW y recuento de plaquetas (PLT). También se incluyó el recuento diferencial relativo de neutrófilos (NEUT%), linfocitos (LINF%), monocitos (MONO%), eosinófilos (EO%), basófilos (BASO%) y los recuentos diferenciales absolutos de neutrófilos (NEUT#), linfocitos (LINF#), monocitos (MONO#), eosinófilos (EO#) y basófilos (BASO#). Así mismo, se calcularon el índice NLR (aplicando el índice NEUT#/LINF#) y el índice PLR (aplicando el índice PLT#/LINF#). Los valores se expresaron empleando el sistema internacional de unidades de medida para hemogramas.

### Criterios de inclusión

Se seleccionaron adultos de entre 18 y 65 años de edad, aparentemente sanos, asistidos en el contexto de campañas de promoción de la salud y prevención, y que cumplieran los criterios establecidos por el Laboratorio Clínico para evitar errores preanalíticos. El grupo fue seleccionado aplicando criterios estrictos, asegurándose de que no presentaran alteraciones significativas en las pruebas analíticas. Se empleó una base de datos previamente seleccionada, que no mostrara alteraciones bioquímicas, y cuyos datos ya habían sido publicados [16]. Para el análisis, se consideraron las variables de sexo y grupo étnico. El criterio aplicado para seleccionar el grupo étnico fue que la persona elegida tuviera al menos un apellido indígena [17] debidamente validado por la Corporación Nacional de Desarrollo Indígena (CONADI) de Chile y por el Servicio Electoral de Chile (Servel).

### Criterios de exclusión

Se excluyó del estudio a la población geriátrica y pediátrica, ya que los individuos de edad avanzada o muy jóvenes podrían presentar variaciones fisiológicas no representativas del intervalo de referencia típico para la población adulta sana. Aquellos datos que no estuvieran dentro de los límites obtenidos en la prueba de Tukey se consideraron valores atípicos y se excluyeron del análisis. Del mismo modo, se excluyeron los parámetros de laboratorio que estaban por debajo o por encima de los límites de decisión clínica (*c* *linical* *d* *ecision* *l* *imits*, CDL), para evitar que individuos con condiciones subyacentes no diagnosticadas sesgaran los resultados. También se eliminaron los registros con datos faltantes o inconsistentes para prevenir errores en el análisis.

### Extracción de la muestra de sangre

Las muestras de sangre se recolectaron en tubos con EDTA-K_3_ de participantes que habían ayunado entre 10 y 12 horas y que no habían realizado actividad física leve o extenuante durante al menos las ocho horas previas. Las muestras se analizaron en un plazo máximo de cuatro horas posteriores a su recolección.

### Análisis estadístico

Los valores de referencia se obtuvieron aplicando el método no paramétrico del intervalo interpercentil recomendado por la IFCC [2] y el Instituto de Normas Clínicas y de Laboratorio (*Clinical and Laboratory Standards Institute*, CLSI). Para cada *cluster*, se calcularon la media y la desviación estándar (DE). Se utilizó la prueba de Tukey para detectar valores atípicos y para establecer los límites inferior [Q1–(1.5 × RIC)] y superior [Q3 + (1.5 × RIC)], siendo RIC el rango intercuartílico (RIC=Q3–Q1).

A continuación, se calcularon los IR mediante el método indirecto no paramétrico basado en rangos interpercentil, que calcula los números de rango de los percentiles 2,5 y 97,5 como límite inferior = 0,025 (n + 1) y límite superior = 0,975 (n + 1), respectivamente. El intervalo de confianza de cada percentil se determinó aplicando la distribución binomial. Las diferencias por sexo se analizaron mediante la prueba U de Mann–Whitney. Un valor p inferior a 0,05 (p<0,05) se consideró estadísticamente significativo.

### Declaración ética

Este estudio fue aprobado por el Comité de Ética de Investigación de la Universidad Católica de Temuco (código: 011601/23) y realizado de acuerdo a los principios de la Declaración de Helsinki (principios éticos para la investigación médica en humanos).

## Resultados

El estudio se realizó en una muestra de 356 personas para establecer los IR de la población general de Chile, con una mediana de edad de 35 años en las mujeres y 36 años en los hombres. Para evaluar posibles diferencias relacionadas con la etnicidad, se emplearon datos de 146 personas que cumplían los criterios para ser identificados como pertenecientes a la etnia mapuche, con una distribución por sexo del 73 % de mujeres y 27 % de hombres, y una mediana de edad de 34 años en el grupo mapuche y en el grupo no mapuche.

Con el fin de establecer IR mediante el método no paramétrico, se inspeccionó visualmente cada parámetro a través de histogramas. Así mismo, se utilizó la prueba de Tukey para identificar y excluir los valores atípicos. En las Tablas 1 y 2 se muestran los límites superior e inferior de los IR calculados, los valores atípicos y la DE para las 21 pruebas analíticas, estratificados por sexo y pertenencia al grupo étnico mapuche. Se observó una baja tasa de exclusión, con un intervalo de entre 1 y 24 valores atípicos en los parámetros estudiados.

**Tabla 1:** Intervalos de referencia de parámetros hematológicos en la población general por género (n=356).

Parámetro	Unidades	Género	IR	Intervalo de confianza (95 %)	Valores atípicos, n	Media	DE	Diferencias hombre-mujer (valor p)
RBC	× 10^12^/L	F	3,84–4,85	(3,34–3,91)–(4,76–5,06)	4	4,363	06,566	< 0,0001^a^
		M	4,27–5,81	(4,19–4,44)–(5,69–6,12)	23	5,78	1,86	
HCT	L/L	F	33,8–40,5	(33–34,6)–(39,9–41,8)	10	37,55	5,108	< 0,0001^a^
		M	38,5–48,2	(36,1–39,1)–(47,5–49,6)	23	49,43	16,13	
HGB	g/L	F	113–137	(110–116)–(135–138)	14	126,7	17,83	< 0,0001^a^
		M	131–169	(124–133)–(166–173)	23	170,9	55,7	
MCH	pg	F	25,9–32,3	(25,3–26,8)–(31,5–33)	13	29,11	4,491	0,3488
		M	26,8–32,9	(16,1–27,8)–(31,7–34,2)	24	33,58	10,9	
VCM	fL	F	78,9–94,7	(76,4–79,6)–(93,3–97,2)	11	86,23	12,8	0,0086^a^
		M	78,1–93,9	(75,8–80)–(92,2–97,4)	23	97,15	31,63	
MCHC	g/L	F	318–353	(313–321)–(353–360)	6	339,3	44,7	< 0,0001^a^
		M	332–362	(324–333)–(360–374)	23	392,2	123,9	
PLT	× 10^9^/L	F	160–393	(127–188)–(365–425)	9	272,3	69,39	< 0,0001^a^
		M	155–367	(100–168)–(337–410)	21	275,4	111	
RDW-DE	fL	F	36,1–46,4	(35,6–37)–(45,4–49)	3	41,26	2,6	0,10
		M	36,5–45,5	(36-36,8)–(44,6–46,7)	23	40,8	2,24	
NLR	n/a	F	0,76–3,06	(0,64–,91)–(2,97–3,37)	10	1,73	0,58	0,0382^a^
		M	0,75–2,93	(0,57–0,83)–(2,54–3,34)	9	1,59	0,55	
PLR	n/a	F	66–211	(44–73)–(204–229)	6	127	37,5	0,0010^a^
		M	50–273	(14–56)–(237–314)	4	119	52,6	
WBC	× 10^9^/L	F	4,07–10,95	(2,17-4,25)–(10,5–11,63)	4	6,889	2,055	0,0978
		M	4,32–11,4	(3,87–4,65)–(10,36–11,97)	15	7,87	3,008	
BASO#	× 10^9^/L	F	0–0,06	(0-0,01)–(0,05-0,07)	1	0,02	0,014	0,0094^a^
		M	0,01–0,07	(0-0,07)–(0,07–0,08)	1	0,031	0,017	
EO#	× 10^9^/L	F	0,03–0,47	(0-0,05)–(0,04–0,05)	12	0,22	0,25	0,0751
		M	0,05–0,57	(0,04–0,06)–(0,49–0,58)	6	0,25	0,16	
NEUT#	× 10^9^/L	F	1,77–6,68	(1,45–2,021)–(5,95–7,28)	4	3,85	1,32	0,0110^a^
		M	1,99–5,81	(1,46–2,15)–(5,24–6,03)	13	3,88	1,58	
LINF#	× 10^9^/L	F	1,21–3,82	(0,96-1,3)–(3,53–4,01)	4	2,21	0,68	0.1421
		M	1,25–3,55	(0,92–1,4)–(3,39–4,16)	9	2,46	1,08	
MONO#	× 10^9^/L	F	0,27–0,84	(0,26–0,31)–(0,81–0,95)	4	0,51	0,15	< 0,0001^a^
		M	0,33–0,9	(0,29–0,36)–(0,82–0,99)	11	0,62	0,21	
BASO%	Porcentaje	F	0–0,9	(0-0,1)–(0,8-1)	5	0,42	0,26	0,0126^a^
		M	0,1–1	(0–0,2)–(0,9–1,1)	5	0,47	0,26	
Linfo%	Porcentaje	F	18,9–50,1	(13,4–20,5)–(45–52)	3	32,92	7,79	0,1421
		M	16,8–53	(14,3–20,6)–(49,5–56,1)	5	35,44	10,8	
EO%	Porcentaje	F	0,5–6,8	(0–0,9)–(6,2–7)	9	3,28	2,16	0,0751
		M	0,6–7,9	(0,5–1)–(7,3–9,4)	5	3,59	2,35	
NEUT%	Porcentaje	F	38–72,4	(35,7–40,7)–(69,5–73,9)	4	55,68	8,76	0,0081^a^
		M	37,7–72	(31,8–39,6)–(67,3–73,8)	7	54,86	12,8	
MONO%	Porcentaje	F	4,5–11,2	(3,4–5,2)–(10,9–12,6)	2	7,69	1,79	< 0,0001^a^
		M	5,5–11,9	(5,3–6,2)–(11,5–12,5)	9	9,0	2,23	

Determinado con la prueba de Mann–Whitney de significación estadística. ^a^Valores con diferencias significativas entre hombres y mujeres, p<0,05. RI, intervalos de referencia; DE, desviación estándar; F, femenino; M, masculino; RBC, recuento de eritrocitos; HCT, hematocrito; HGB, hemoglobina; VCM, volumen corpuscular medio; MCH, hemoglobina corpuscular media; MCHC, concentración de hemoglobina corpuscular media; PLT, recuento de plaquetas; RDW, amplitud de distribución eritrocitaria; NLR, índice neutrófilo-linfocito; n/a, no aplicable; PLR, índice plaqueta-linfocito; WBC, recuento de leucocitos; BASO, basófilos; EO, eosinófilos; NEUT, neutrófilos; LINF, linfocitos; MONO, monocitos; %, recuento relativo; #, recuento absoluto.

**Table 2:** Reference intervals of hematological parameters by ethnicity (n=146).

Parámetro	Unidades	Etnicidad	IR	Intervalo de confianza (95 %)	Valores atípicos, n	Media	SD	Diferencias mapuche-no mapuche (valor p)
RBC	× 10^12^/L	MP	3,94–5,6	(3,47–4,16)–(5,54–5,83)	20	5,413	2,144	< 0,0001^a^
		NMP	3,89–5,06	(3,75–3,94)–(4,94–5,27)	1	4,353	0,33	
HCT	L/L	MP	32,6–46,9	(29,7–33,8)–(46,9–48,6)	21	45,86	18,29	< 0,0001^a^
		NMP	33–41,6	(31–34)–(41,3–41,7)	4	36,98	2,553	
HGB	g/L	MP	103–165	(98–110)–(160–174)	21	155,4	62,4	< 0,0001^a^
		NMP	106–146	(103–110)–(143–148)	4	125,6	11,26	
MCH	pg	MP	25,7–31,3	(25–26,2)–(31,4–32,3)	24	33,07	12,55	0,2265
		NMP	25,8–32,3	(25,8–26,1)–(31,5–33,3)	11	28,92	2,378	
VCM	fL	MP	76,7–94,9	(76–7)–(91,3–93,9)	22	97,64	36,99	0,5297
		NMP	78,8–94,6	(74,2–78,9)–(92,9–97,4)	7	85,17	5,5	
MCHC	g/L	MP	319–358	(308–325)–(355–366)	21	389,4	145	0,5707
		NMP	318–358	(315–321)–(354–362)	6	339,2	11,5	
WBC	× 10^9^/L	MP	4,06–9,48	(3,51–4,34)–(10,21–11,76)	11	7,74	3,48	0,2059
		NMP	4,06–10,31	(2,17–4,19)–(9,45–11,1)	2	6,696	1,784	
RDW-DE	fL	MP	35,9–47,2	(35,6–37,5)–(44,9–46,1)	19	47,57	18,46	0,9527
		NMP	36,4–45,5	(35,6–37)–(45,4–47,2)	3	41,29	2,776	
PLT	× 10^9^/L	MP	175–458	(160–192)–(425–466)	11	305,3	127,6	0,0615
		NMP	171–383	(162–181)–(355–397)	7	265,5	67,48	
NLR	n/a	MP	0,88–3,26	(0,6–0,92)–(2,84–3,76)	7	1,77	0,61	0,3520
		NMP	0,69–3,41	(0,64–0,8)–(2,78–3,47)	9	1,70	0,62	
PLR	n/a	MP	72–224	(64–84)–(209–248)	9	132	39,2	0,1944
		NMP	57–229	(44–69)–(211–248)	6	127	44,4	
BASO%	Porcentaje	MP	0–1	(0-0,2)–(0,8–1)	9	0,48	0,34	0,2168
		NMP	0–0,9	(0–0,9)–(0,8–1)	4	0,4	0,25	
EO%	Porcentaje	MP	0,7–6,6	(0,1–0,9)–(5,4–7,1)	12	3,48	3,06	0,7048
		NMP	0,5–6,9	(0–0,8)–(6,1–7)	7	3,21	2,3	
NEUT%	Porcentaje	MP	39,9–77,5	(32,2–41,4)–(69,3–78,8)	7	59,38	19,25	0,2772
		NMP	37,4–71,8	(36–39,7)–(69,7–73,9)	3	55,15	9,48	
LINF%	Porcentaje	MP	16–50,3	(11,9–18,9)–(46,6–53,6)	4	34,41	12,39	0,8114
		NMP	16,3–50,1	(13,4–20)–(49,5–54)	3	33,17	8,83	
MONO%	Porcentaje	MP	4,2–11,5	(3,8–5,1)–(10,4–12,4)	10	8,42	11,96	0,0735
		NMP	5,2–12	(3–5,3)–(11,2–13)	0	8,07	1,93	
BASO#	× 10^9^/L	MP	0–0,06	(0–0,1)–(0,06–0,07)	8	0,032	0,023	0,2131
		NMP	0–1	(0–1)–(1–2)	0	0,025	0,014	
LINF#	× 10^9^/L	MP	0,03–0,45	(0,01–0,06)–(0,37–0,46)	13	0,23	0,21	0,8772
		NMP	0,03–0,5	(0-0,05)–(0,43–0,5)	9	0,21	0,2	
EO#	× 10^9^/L	MP	1,7–6,78	(1,32–1,97)–(6,18–6,78)	7	4,09	1,94	0,1738
		NMP	1,8–6,02	(1,46–2,01)–(5,68–6,59)	7	3,73	1,4	
NEUT#	× 10^9^/L	MP	1,09–3,41	(0,8–1,34)–(2,95–3,43)	8	2,27	0,95	0,7108
		NMP	1,04–3,35	(0,29–1,21)–(0,29–4,01)	3	2,18	0,71	
MONO#	× 10^9^/L	MP	0,26–0,86	(0,2–0,32)–(0,76–0,87)	8	0,57	0,31	0,2181
		NMP	0,31–0,95	(0,27–0,32)–(0,81–10,95)	2	0,53	0,16	

Determinado con la prueba de Mann–Whitney de significación estadística. ^a^Valores con diferencias significativas entre hombres y mujeres, p<0,05. RI, Intervalos de referencia; DE, desviación estándar; F, femenino; M, masculino; RBC, recuento de eritrocitos; HCT, hematocrito; HGB, hemoglobina; VCM, volumen corpuscular medio; MCH, hemoglobina corpuscular media; MCHC, concentración de hemoglobina corpuscular media; PLT, recuento de plaquetas; RDW, amplitud de distribución eritrocitaria, NLR, índice neutrófilo-linfocito; n/a, no aplicable; PLR, índice plaqueta-linfocito; WBC, recuento de leucitos; BASO, basófilos; EO, eosinófilos; NEUT, neutrófilos; LINF, linfocitos; MONO, monocitos; %-recuento relativo; #, recuento absoluto.

La Tabla 1 muestra los IR obtenidos para la población adulta general, separados por sexo (hombres y mujeres). En la Tabla 2 se presenta el análisis según etnicidad (mapuche y no mapuche). Dado que el tamaño de la muestra de los individuos del grupo mapuche era de 146, no se pudo segregar el análisis por sexo, dado que no se hubiera alcanzado el número mínimo requerido de 120 datos por cada grupo (hombres y mujeres) recomendado por la IFCC.

Observamos diferencias estadísticamente significativas entre los hombres y las mujeres en RBC, HCT, HGB, VCM, MCHC, PLT, NLR y PLR, pero no en MCH, WBC, EO%, LINF%, EO# y LINF#. En general, los hombres suelen presentar valores más altos en los hemogramas, excepto en VCM, PLT, WBC y NEUT#. Los valores de los parámetros de la fórmula leucocitaria diferencial, tanto absolutos como relativos, fueron muy similares en los dos grupos de estudio.

**Figura 1:**
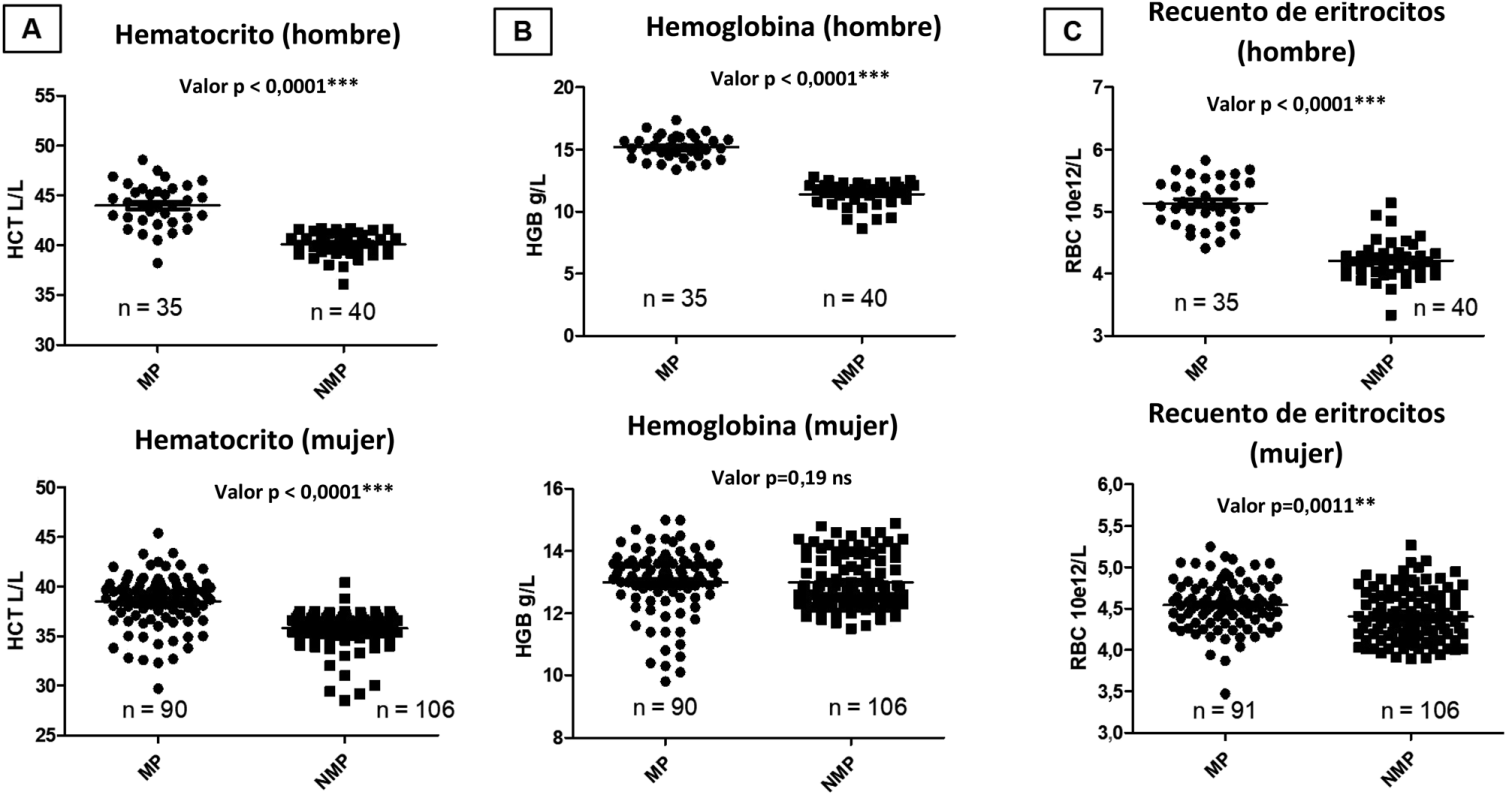
Distribución de frecuencias y significación estadística según el origen étnico y el sexo de los parámetros HCT, HGB y RBC. (A) Hematocrito (hombre y mujer). (B) Hemoglobina (hombre y mujer). (C) Conteo eritrocitario (hombre y mujer). PM, población mapuche; PNM, población no-mapuche.

En los IR obtenidos en individuos mapuches en comparación con los no mapuches, se observaron diferencias estadísticamente significativas en RBC, HGB y HCT (p<0,0001). La mayoría de los parámetros presentaron valores más altos en la población mapuche. Para confirmar que dichos resultados no estaban relacionados con el tamaño de los grupos por sexo, dado que en el grupo mapuche el 72 % eran mujeres y el 28 % eran hombres, mientras que, en el grupo no mapuche, el 73 % eran mujeres y el 27 % eran hombres, se analizaron tres parámetros desagregados por sexo (Figura 1). Se observaron diferencias significativas en el HCT en las mujeres (90 mapuche y 106 no-mapuche) con un valor p de <0,0001 (Figura 1), lo que corrobora que las diferencias significativas entre hombres y mujeres no estaban relacionadas con el tamaño de los grupos.

En el análisis de los hombres (mapuche vs. no mapuche, n=35 y n=40 respectivamente), se observaron diferencias estadísticamente significativas (p<0,001, Figura 1), mostrando que los hombres mapuche tienden a presentar valores más elevados de HCT, lo que concuerda con los valores de RBC (en ambos sexos; mapuche vs. no mapuche) y para la hemoglobina en los hombres, pero no en las mujeres (valor p>0,05; Figura 1).

Aunque los valores de PLT fueron más altos en el límite superior en la población mapuche (458 × 10^9^/L vs. 383 × 10^9^/L respectivamente, Tabla 2), esta diferencia no alcanzó significancia estadística (valor p=0,0615).

Al comparar los IR empleados por el Laboratorio Clínico UC Temuco (datos no mostrados), se observó una mayor similitud con los obtenidos en los individuos no mapuches, especialmente en los parámetros de RBC (4,4–5,6 × 10^12^/L); HCT (37,5–47,5 L/L); HGB (125–158 g/L) y PLT (150–450 × 10^9^/L), lo que demuestra que esta diferencia no solo se observa en este grupo étnico, sino también en otras poblaciones que son estudiadas por los fabricantes de reactivos.

## Discusión

Los estudios realizados para establecer los IR de parámetros hematológicos en la población latinoamericana son escasos. Chile no es la excepción, ya que únicamente se han publicado dos artículos al respecto. En el primer estudio, se analizó una muestra aleatoria de militares voluntarios de sexo masculino expuestos a una altitud elevada (3.550 m) en Putre (Chile) [14]. En el otro estudio, se evaluaron los reticulocitos en una población pediátrica [13]. Los IR obtenidos de la población general en este estudio (Tabla 1) indican que la mayoría de las diferencias estaban asociadas al sexo. Las mujeres presentaron valores más altos de VCM, PLT y NLR que los hombres. Por el contrario, se observaron concentraciones más altas de HGB, RBC, HCT y PLR (límite superior) en los hombres. Estos resultados subrayan la importancia de segregar los IR del hemograma por sexo.

Además, al analizar estos resultados conjuntamente con los de otros autores, observamos que los IR obtenidos en nuestro estudio para RBC, VCM, MCH y MCHC son similares a los documentados por Rosenfeld y col. [18], Fernández y col [19] y Adeli y col [20], aunque en este último se incluyeron grupos diferentes y con rangos de edad distintos. En estos estudios, valores más elevados de RBC (límite superior, mujeres) han sido observados en comparación a nuestro trabajo. Del mismo modo, se obtuvieron IR más altos en los hombres, en los parámetros RBC, HGB y HCT, lo que concuerda con los estudios realizados en Irlanda [21], Bélgica [22], España [23] y Turquía [24]. Los niveles más bajos de hemoglobina observados en las mujeres también coinciden con los hallazgos de otros estudios [25]. No se observaron diferencias significativas en VCM y MCH entre hombres y mujeres, lo que también coincide con los estudios anteriormente mencionados.

Respecto al recuento de plaquetas, en general, existe cierta similitud entre los estudios, a excepción del realizado por Rosenfeld y col. [18], que obtuvieron valores más bajos en las mujeres. Además, documentaron valores de HCT (en los dos sexos) y HGB (en las mujeres) más elevados, y valores de WBC más bajos que en nuestro estudio [18]. Por otro lado, Sáenz y col. [26], que analizaron una población altoandina ecuatoriana, establecieron un intervalo del recuento leucocitario de 6,7–7 × 10^9^/L, lo que es significativamente inferior a los niveles observados en nuestro estudio. Sin embargo, las condiciones de altitud de los individuos en ambos estudios fueron notoriamente diferentes. El recuento leucocitario relativamente elevado de nuestro estudio, comparado con otros, se podría deber a factores como la etnicidad, variaciones circadianas, embarazo, estrés, ejercicio y/o el uso de medicamentos [27], 28]. Estas diferencias fueron particularmente evidentes el comparar los datos con los publicados por Islam y col. [21], Florin y col. [22]. y Arbiol-Roca y col. [23]. Con respecto a los datos leucocitarios (Tabla 1), se observaron diferencias mínimas con los datos reportados por el Laboratorio Clínico UC Temuco.

Las variaciones en los IR por sexo podrían estar asociadas a diferencias en el sistema endocrino. Existe evidencia de que la testosterona induce la eritropoyesis al aumentar la eritropoyetina y al suprimir la hepcidina [29]. La hepcidina es un péptido que actúa bloqueando el flujo de hierro celular. Además, se ha demostrado que los estrógenos afectan directamente al factor de transcripción GATA1, lo que produce un incremento significativo en la apóptosis de células eritroides [30]. Asimismo, las fluctuaciones en los hormonas esteroides femeninas durante el ciclo menstrual provocan cambios en los parámetros hematológicos como HCT, HGB, el recuento de neutrófilos y el recuento de eosinófilos [31]. Estas modificaciones estimulan la médula ósea, induciendo el flujo sanguíneo de eritrocitos inmaduros a la sangre periférica. Combinado con la prevalencia relativamente alta de anemia por déficit de hierro en las mujeres durante la menstruación, estos factores explicarían las diferencias en los IR de estos parámetros por sexo [32]. Por otro lado, la masa muscular de las mujeres suele ser entre un 25 y un 40 % inferior a la de los hombres [33], lo que resulta en una mayor demanda de oxígeno y flujo sanguíneo en estos. De este modo, un mayor índice de masa corporal está asociado a niveles más elevados de WBC, RBC, HGB, HCT y PLT en niños y adolescentes [34]. También se han tomado en cuenta otros factores, como el ejercicio de alta intensidad, que puede incrementar la concentración de HGB, HCT y RBC, pudiendo reducir el volumen total de sangre [35], aunque este factor fue considerado en los criterios de inclusión y exclusión de nuestro estudio.

Con respecto al RDW no se observaron diferencias estadísticamente significativas en relación al sexo o el grupo étnico. El IR obtenido fue muy similar al empleado actualmente por el Laboratorio Clínico UC Temuco (RDW-DE 37–46  fL); aunque no pudimos comparar estos parámetros con los publicados por otros autores, ya que solo presentamos el RDW como desviación estándar (RDW-DE). La principal aplicación clínica del RDW, junto con otros parámetros como el VCM, ha sido el estudio de la anemia por déficit de hierro y la talasemia β, así como otros déficits nutricionales que causan anemia megaloblástica como el déficit de ácido fólico o de vitamina B12 [36]. Sin embargo, algunos estudios demuestran que el RDW también puede encontrarse alterado en sujetos con diferentes patologías cardíacas, diabetes, enfermedad renal, así como en pacientes críticos [37].

Además, calculamos el NLR y el PLR, que indican el estado inflamatorio del individuo [6], ya que están asociados a diferentes procesos como los efectos secundarios posteriores a la trombólisis en pacientes con accidente cerebrovascular isquémico agudo [38]. Actualmente, los estudios realizados para establecer IR para estos índices son escasos. Por ejemplo, en la población china, el IR de NLR fue de 0,43–2,75 para los hombres y de 0,37–2,87 para las mujeres, mientras que el IR de PLR fue de 36,63–149,13 para los hombres y de 43,36–172,68 para las mujeres [39]. En Corea del Sur, el IR del NLR fue similar al de nuestro estudio, con una media de 1,7 para los hombres (intervalo de edad de entre 20 y 49 años) y una media de 1,6 para las mujeres. Únicamente se observó mayor similitud en el PLR en el rango de edad superior a los 70 años [40]. En la población iraní, se han establecido un NLR y PLR media de 1,70 ± 0,70 y 117,05 ± 47,73, respectivamente [41]. En la población nigeriana, se obtuvieron valores de 2,8 (1,2–4,4) para NLR, y 137 (75–199) para PLR [42], siendo este último superior al observado en nuestro estudio.

Aunque el concepto y la utilidad de los IR son sencillos, su establecimiento resulta complejo, ya que existen factores que dependen de las características de los individuos, los criterios de inclusión y exclusión, el equipo y metodologías empleados para su cálculo [24]. Es por ello que su determinación requiere la aplicación de procesos preanalíticos y analíticos rigurosamente normalizados, con métodos estadísticos apropiados y muestras representativas de la población. Algunos estudios presentan un tamaño muestral limitado, como los realizados por Arbiol-Roca y col. con 213 datos [23], Islam y col. con 132 [21], Molina y col. con 135 [28], y Fernández y col. con 250 datos [19]. Sin embargo, cabe mencionar que todos estos estudios seguían incluyendo muestras de tamaño superior al recomendado por la IFCC para los análisis no paramétricos (120 datos). Por otro lado, algunos estudios emplearon tamaños muestrales significativamente mayores, como el de Rosenfeld y col. con 8.952 [18] y Hollowell y col. con 26.372 [43]. Con respecto a las diferencias analíticas, se emplearon equipos diferentes, aunque la mayoría de los autores utilizaron el analizador hematológico Sysmex XE-2100 [22], 23], 26], 28] y el ADVIA 2120i [21]. Del mismo modo, para el análisis estadístico, algunos investigadores emplearon también la prueba de Tukey para excluir los valores atípicos [22], 23], mientras que otros calcularon los IR a partir de X ± 2SD (media ± 2 desviaciones estándar), lo que reduce la precisión de los intervalos. En nuestro caso, empleamos el método del intervalo interpercentil, siguiendo la recomendación de la IFCC [2].

Si bien los IR se determinan en individuos predominantemente caucásicos [44], Latinoamérica es una región multiétnica y multicultural, con 671 pueblos indígenas reconocidos [45]. A través de marcadores del genoma mitocondrial, los estudios de genética poblacional han mostrado que la población chilena tiene un origen genético principalmente amerindio [46]. Además, los factores migratorios en Chile están en aumento y también la visibilidad de los pueblos indígenas en las ciudades chilenas ha seguido aumentando en las últimas décadas [47], principalmente a causa de los llamados “factores de atracción”, como un mayor acceso a bienes y servicios, el desarrollo y extracción de recursos naturales y desequilibrios territoriales [45]. Por esta razón, estudiamos la variable de etnia mapuche, observando diferencias estadísticamente significativas en RBC, HGB y HCT (p<0,0001), con respecto a la población no mapuche. Aunque no se observaron diferencias significativas en otros parámetros, se observó que los valores medios tendían a ser más elevados en la población mapuche en la gran mayoría de los parámetros estudiados.

Otros estudios en diferentes grupos étnicos (caucásicos, negroide y asiáticos) han evidenciado diferencias importantes, como un IR significativamente menor en asiáticos en HCT, HGB, HCM, MCHC y el volumen plaquetario medio [48]. Los individuos de etnia negroide presentan un IR significativamente menor en HCT, HGB, HCM, MCHC, colesterol total, triglicéridos y WBC, frente a los individuos de origen caucásico. Lim y col [48]. observaron que los hispanos presentan un IR inferiore en HCM y MCHC [48]. Cheng y col. [49] observaron porcentajes más elevados en células mononucleares y linfocitos en la población negroide, con una menor proporción de granulocitos en todas las edades en ambos sexos. Los valores de HCT, MCHC, MCH y HGB fueron también más bajos para todas las edades y en ambos sexos [49].

Respecto a las limitaciones del estudio, el reducido tamaño de la muestra de población mapuche no permitió realizar un análisis segregado por sexo. Además, los criterios de inclusión en este grupo étnico establecían la presencia de al menos un apellido mapuche, por lo que, probablemente, se incluyeron individuos de origen mestizo en el análisis. Del mismo modo, teniendo en cuenta los patrones migratorios internos de Chile, que suelen derivar en cambios en el estilo de vida, sería necesario realizar estudios adicionales para analizar directamente los IR en las distintas comunidades étnicas en esta y otras regiones.

En conclusión, los IR se han establecido predominantemente en poblaciones caucásicas, por lo que es importante destacar que Latinoamérica es una región multiétnica y multicultural. El presente estudio es el primero en establecer IR para parámetros hematológicos en población adulta chilena y el grupo étnico mapuche en Chile. Algunos de los parámetros presentan mayor precisión y variabilidad, dependiendo de la población analizada, su ubicación geográfica y el sexo, mientras que otros varían según el origen étnico. Existen importantes diferencias en relación a algunos parámetros, como RBC, HCT, y HGB, entre la población mapuche y otras empleadas por el Laboratorio Clínico UC Temuco. Por lo tanto, es importante recalcar que la adaptación de los IR tiene un impacto directo en la calidad de la asistencia médica, apoyando diagnósticos más precisos , y disminuyendo el riesgo de errores de interpretación, como el subdiagnóstico de la anemia o la sobreestimación de alteraciones hematológicas. Así mismo, permitiría una mejor adaptación de los tratamientos, ya que los límites de referencia reflejan las características fisiológicas de cada grupo. En el contexto de las poblaciones indígenas o de diferentes grupos étnicos, el empleo de IR basados en datos de población caucásica podría derivar en la administración de tratamientos inadecuados. Esto también tiene un impacto en las políticas de salud pública, permitiendo una evaluación más precisa de la salud de la población y una mejor planificación de las intervenciones. Esto resulta especialmente relevante en Latinoamérica, donde se deben tener en cuenta las diferencias étnicas para evitar desigualdades sanitarias. Además, resalta la importancia de planificar estudios de validación para garantizar la extrapolación adecuada de los datos y de llevar a cabo estudios multicéntricos que tengan en cuenta estas variables en poblaciones concretas. Finalmente, estos datos proporcionan una orientación valiosa para futuros estudios y para la práctica clínica en nuestra región, al ofrecer evidencia contextualizada que puede influir en la toma de decisiones clínicas.
